# The expression and function of miR-424 in infantile skin hemangioma and its mechanism

**DOI:** 10.1038/s41598-017-10674-7

**Published:** 2017-09-19

**Authors:** Lili Yang, Jun Dai, Fan Li, Henghui Cheng, Dan Yan, Qiurong Ruan

**Affiliations:** 1 Institute of Pathology, Tongji Hospital, Tongji Medical College, Huazhong University of Science and Technology, Wuhan, 430030 China; 2 Cancer Biology Research Center, Tongji Hospital, Tongji Medical College, Huazhong University of Science and Technology, Wuhan, 430030 China; 3 Department of Urology of Wuhan Central Hospital, Wuhan, 430014 China; 4 Department of Pathology, Medical College, Wuhan University of Science and Technology, Wuhan, 430065 China

## Abstract

Infantile hemangioma is the most common benign tumor in infants. Many studies have confirmed that basic fibroblast growth factor (bFGF) and its key receptor FGFR1 are highly expressed in hemangioma. Moreover, several miRNAs can regulate angiogenesis. In this regard, miR-424 often plays a role as tumor suppressor gene. This study was designed to investigate the mechanism of miR-424 in infantile skin hemangioma. Our results showed low expression of miR-424 in infantile skin hemangioma tissues, and that miR-424 overexpression downregulated FGFR1 expression in hemangioma-derived endothelial cells, while miR-424 inhibition upregulated FGFR1 expression. Luciferase reporter analysis confirmed that FGFR1 was a target gene of miR-424. CCK-8, flow cytometry, transwell migration and tube formation assays demonstrated that miR-424 overexpression inhibited cell proliferation, migration and tube formation, at least in part by blocking the bFGF/FGFR1 pathway. In contrast, miR-424 inhibition significantly enhanced these functions. Furthermore, miR-424 overexpression significantly inhibited ERK1/2 phosphorylation, whereas miR-424 inhibition enhanced ERK1/2 phosphorylation. In conclusion, miR-424 could suppress the bFGF/FGFR1 pathway, thereby inhibit ERK1/2 phosphorylation, and thus inhibit cell proliferation, migration and tube formation capabilities and the development of infantile skin hemangioma.

## Introduction

Infantile hemangioma (IH) is the most common benign vascular lesion in infants and young children, and it is characterized by the abnormal growth of endothelial cells. Typical IH lesions appear in the first few weeks after birth, rapidly proliferate in 6–10 months, and slowly degrade after 5–10 years^1^. Bauland *et al*. showed that the maximum size of IHs was reached at 8 months while involution started at a median age of 2 years and was completed at a median age of 4 years^2^. Couto *et al*. also estimated that involution ceased at a median age of 36 months and that 92 percent of tumors completed involution by 48 months^3^. However, 40–80% of IHs leave permanent scars or a large amount of adipose tissue after tumor regression, especially in facial lesions, which can cause disfigurement^4^. The incidence of IHs ranges from 1.1% to 2.6%, with the highest incidence being 10–12%^5^. IHs occur mainly in females (the male-to-female ratio is 1: 3), preterm children, and children with placental abnormalities during pregnancy^6^. Eighty percent of hemangiomas are located in the head and neck region, and most of them are relatively small, sporadic, independent, located in the skin, and present few clinical problems and therefore do not need treatment^7,8^. However, nearly twenty percent of IH patients have multiple lesions. These lesions can grow invasively and/or be located on relatively important tissues, and approximately ten percent of hemangiomas grow quickly. In these cases, the tumors can cause many problems and even disrupt normal tissue or threaten the life of the patient^9,10^. At present, although the history and progression of these lesions are well understood, their etiology and the exact mechanism of their occurrence and spontaneous degradation remain unclear. A better understanding of hemangioma pathogenesis will provide innovative ideas for exploration of more effective treatment strategies.

Basic fibroblast growth factor (bFGF, also called FGF2) is one of the most effective and specific angiogenic factors, and it can stimulate the proliferation of endothelial cells and muscle cells. bFGF can also stimulate fibroblast proliferation and migration, and the generation of proteoglycans, collagen, fibronectin, hyaluronic acid and integrins, all of which play important roles in angiogenesis^11–13^. bFGF mainly exerts its functions by binding to its key receptor, fibroblast growth factor receptor 1 (FGFR1), on the surface of target cells to induce its autophosphorylation and thereby activates various functional proteins and participates in many signal transduction pathways that control cell proliferation, differentiation, survival, and angiogenesis^14,15^. Many reports have confirmed that overexpression of bFGF paralleled with the growth of proliferating hemangiomas, suggesting that the bFGF/FGFR1 signaling pathway is not only involved in hemangioma formation but is also closely related to the proliferation and involution of hemangiomas and can be used as the primary indicator of vascular tumor growth^16–18^.

MicroRNAs (miRNAs) are a class of non-coding, small-molecule, single-stranded RNAs (generally 18 to 22 nucleotides in length) that are highly evolutionarily conserved. They are involved in the regulation of various pathophysiological processes, such as cell proliferation, differentiation and apoptosis^19,20^. miRNAs play important roles in the development and progression of many tumors by participating in gene regulation^21,22^. By interacting with the 3′UTR of mRNAs, miRNAs can regulate the expression of many genes simultaneously and usually affect each signaling pathway at multiple levels^23,24^. Many recent studies have also revealed how miRNAs regulate angiogenesis^25–29^. The miR-15/107 family of genes has been reported to regulate gene expression involved in cell division, metabolism, stress response, and angiogenesis in vertebrate species and is implicated in many human diseases^30^. Thereinto, the miR-15 superfamily, comprising miR-15a, miR-15b, miR-16, miR-195, miR-322, miR-424, miR-457, and miR-497, has been hypothesized to evolve in the common ancestors of vertebrates and is highly conserved^31–33^. As miR-424 is currently a fascinating topic of study, it was selected for our study to investigate its expression, role and mechanism in infantile skin hemangioma. MiRbase shows that miR-424–5p is located on human chromosome Xq26.3^34^. Many studies have reported that miR-424-5p acts as a tumor suppressor gene^35–38^. However, miR-424-5p is usually upregulated in pancreatic cancer and exerts its role by modulating the ERK1/2 signaling pathway^34^. In addition, Nakashima *et al*. noted that miR-424 was downregulated in senile hemangioma, suggesting that miR-424 also acted as a potential inhibitory miRNA in senile hemangioma^39^. Therefore, we predicted that miR-424 plays a tumor suppressor role in infantile hemangioma and that FGFR1 may be its target.

In this study, our aim was to investigate the expression, role and mechanism of miR-424 in infantile skin hemangioma. Our study indicated that miR-424 may reduce FGFR1 expression, suppress the bFGF/FGFR1 pathway, thereby inhibit ERK1/2 phosphorylation, and thus inhibit the cell proliferation, migration and tube formation capabilities and the development of infantile skin hemangioma.

## Results

### miR-424 was downregulated in infantile skin hemangioma tissues

**Figure 1:**
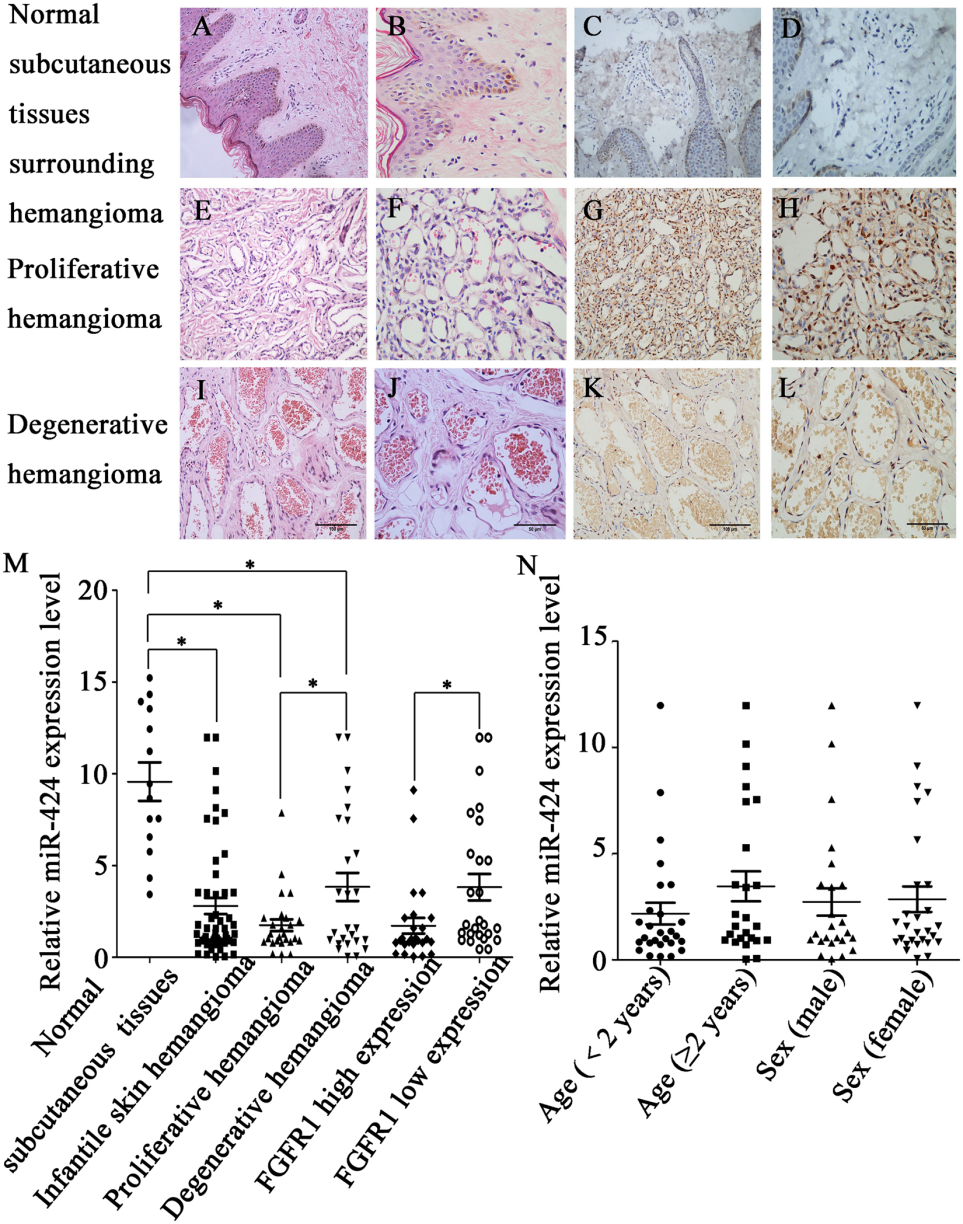
Expression of miR-424 and FGFR1 in infant skin hemangioma tissues and their correlation. (**A**,**B**) Normal subcutaneous tissue surrounding hemangiomas (**A**), ×200; (**B**), ×400). (**C**,**D**) The expression level of FGFR1 in normal subcutaneous tissue surrounding hemangiomas (**C**, × 200; **D**, × 400). (**E**,**F**) Proliferative hemangioma tissues (**E**), ×200; (**F**), ×400). (**G**, **H**) FGFR1 was relatively highly expressed in proliferative hemangioma tissues (**G**), ×200; (**H**), ×400). (**I**,**J**) Involuting hemangioma tissues (**I**), ×200; (**J**), ×400). (**K**,**L**) FGFR1 was expressed at a relatively low level in involuting hemangioma tissues (**K**), ×200; (**L**), ×400). (**M**) The association between miR-424 expression and proliferative phase, involuting phase and the expression of FGFR1; (**N**) The relationship between miR-424 expression and clinical characteristics such as age and sex. (**A**,**B**,**E**,**F**,**I**,**J**) HE staining; (**C**,**D**), (**G**,**H**,**K**,**L**): Immunohistochemical staining; (**M**,**N**): statistical analysis, *P < 0.05 indicates statistically significant differences.

According to the WHO criteria, 52 cases of infantile skin hemangioma were divided into proliferative hemangiomas and involuting hemangiomas via microscopic evaluation (26 cases, respectively) (Fig. 1E,F,I,J). Figure 1A and B showed the normal subcutaneous tissue surrounding the hemangiomas. Real-time PCR was used to detect the expression level of miR-424 in infantile skin hemangioma tissues and normal subcutaneous tissue surrounding hemangiomas. The results revealed low expression of miR-424 in infantile skin hemangioma tissues compared with the normal subcutaneous tissue surrounding hemangiomas, and the miR-424 expression in proliferative hemangiomas was lower than that in involuting hemangiomas (*P* < 0.05) (Fig. 1M).

Many studies have reported that bFGF and its receptor FGFR1 are highly expressed in hemangiomas. To confirm this statement and explore the relationship between miR-424 and FGFR1, we examined the FGFR1 expression in infant skin hemangioma tissues using immunohistochemistry. Based on the staining intensity, FGFR1 expression was divided into four levels (0, 1, 2 and 3): 0, negative expression of FGFR1 in infantile skin hemangioma tissues; 1, weakly positive expression of FGFR1; 2, moderately positive expression of FGFR1; and 3, strongly positive expression of FGFR1. Levels 0 and 1 were categorized as low FGFR1 expression, while levels 2 and 3 were considered to represent high FGFR1 expression. Our results showed that FGFR1 was expressed at a low level in normal subcutaneous tissue surrounding hemangiomas, while the expression rate of FGFR1 was 48.08% (25/52) in infantile skin hemangioma tissues, of which 73.08% (19/26) in the proliferative phase and 23.08% (6/26) in the involuting phase, indicating that FGFR1 expression in infantile skin hemangioma tissues was significantly higher than that in normal subcutaneous tissue surrounding hemangioma and that FGFR1 expression was higher in the proliferative phase than in the involuting phase (Fig. 1C,D,G,H,K,L). Statistical analysis showed that miR-424 was expressed at low levels in tissues with relatively high FGFR1 expression, while miR-424 exhibited a high expression level when FGFR1 was expressed at a relatively low level (Fig. 1M), suggesting that the miR-424 expression in infantile skin hemangioma tissues was negatively correlated with FGFR1.

In addition, we analyzed the relationship between the expression of miR-424 in infantile skin hemangioma tissues and clinical characteristics, such as age and sex (Fig. 1N). Our results revealed no significant correlation between the expression level of miR-424 and the patients’ age or sex.

**Figure 2:**
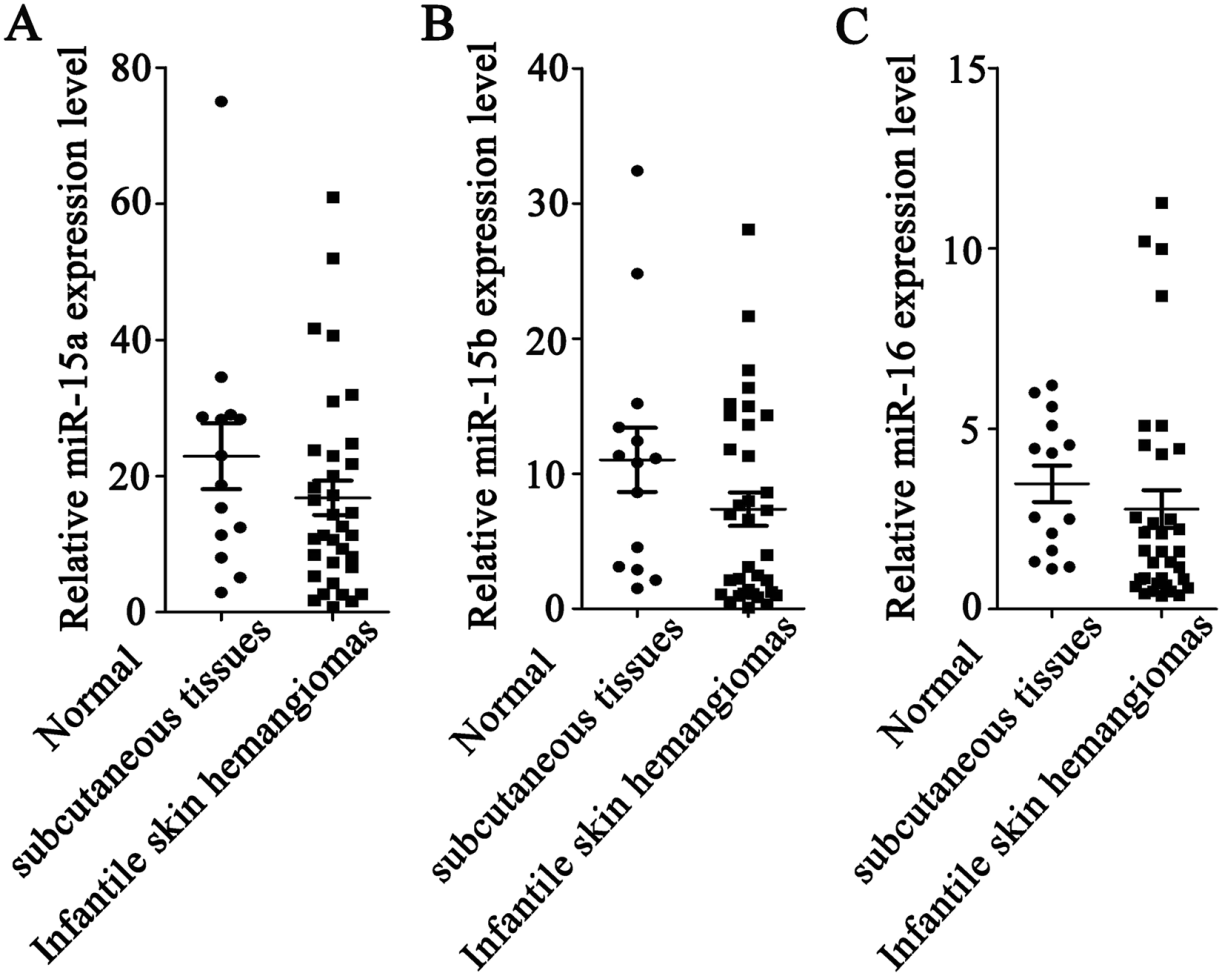
Expression of miR-15a, miR-15b and miR-16 in infantile skin hemangioma tissues and normal subcutaneous tissue surrounding hemangioma. The expression of (**A**) miR-15a, (**B**) miR-15b, and (**C**) miR-16.

Furthermore, the expression level of miR-15a, miR-15b and miR-16 were also detected in infantile skin hemangioma tissues and normal subcutaneous tissue surrounding hemangiomas using real-time PCR (Fig. 2). As we envisioned, there were no significant difference in the expression of miR-15a, miR-15b and miR-16 between infantile skin hemangioma tissues and normal subcutaneous tissue surrounding hemangiomas.

### miR-424 regulated the expression of FGFR1 in HemECs

**Figure 3:**
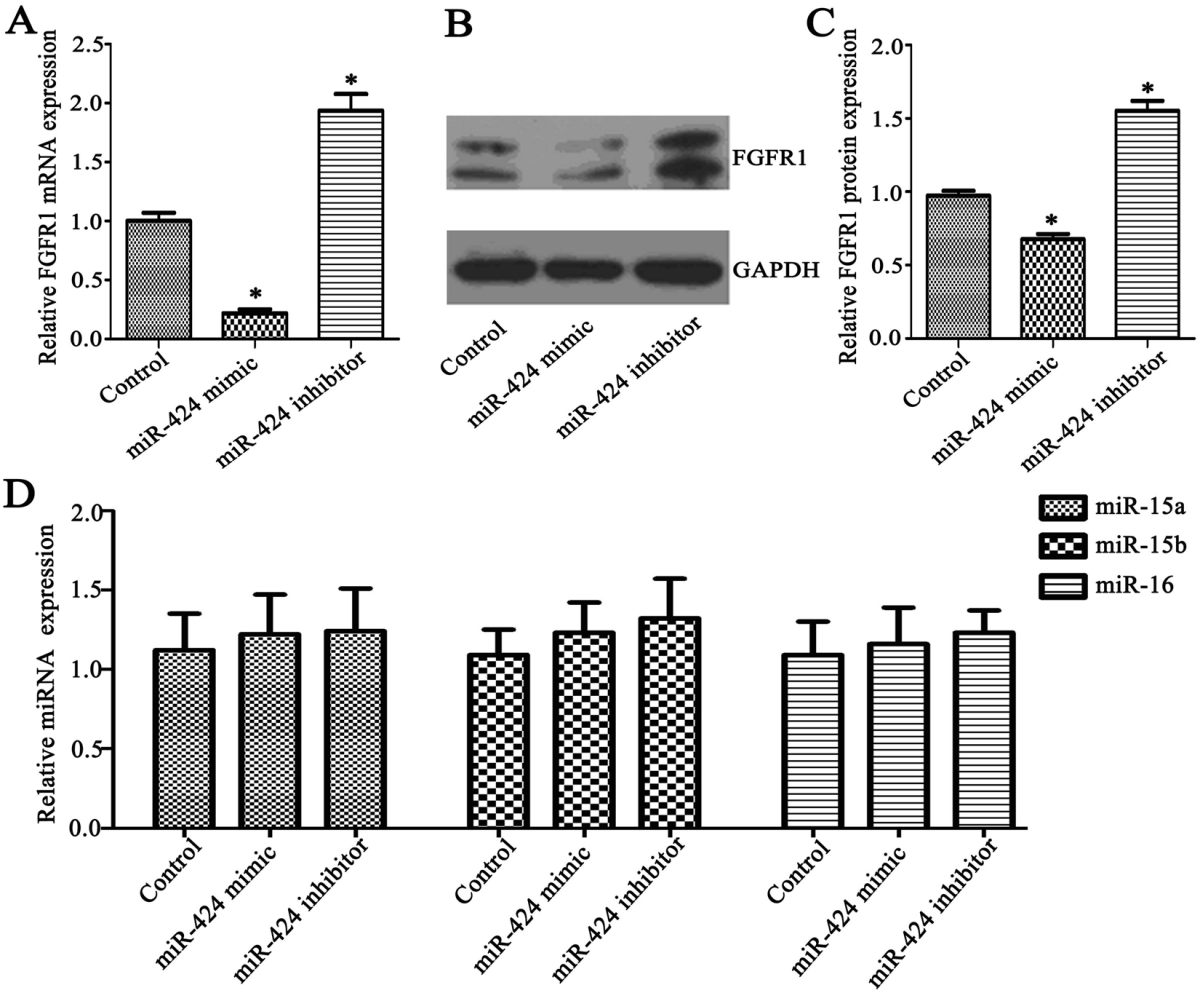
miR-424 regulated the expression of FGFR1 in HemECs. (**A**) Changes in FGFR1 mRNA expression were detected with real-time PCR after HemECs were transfected with miR-424 mimic or inhibitor. (**B**,**C**) Changes in FGFR1 protein expression were detected using western blot analysis after HemECs were transfected with miR-424 mimic or inhibitor. (**D**) Expression of miR-15a, miR-15b and miR-16 in HemECs after transfection with miR-424 mimic or inhibitor. Three replicates were performed for each sample. *P < 0.05 indicated statistically significant differences between the experimental group and control group.

To investigate whether miR-424 can regulate the expression of FGFR1 in hemangioma-derived endothelial cells (HemECs), the cells were transfected with miR-424 mimic or miR-424 inhibitor, and HemECs transfected with control mimic or control inhibitor were used as negative controls. Real-time PCR showed that the FGFR1 mRNA level in miR-424 mimic-transfected HemECs was significantly lower than that in control mimic-transfected HemECs (*P* < 0.05), whereas the FGFR1 mRNA level was significantly higher in miR-424 inhibitor-transfected HemECs than that in control inhibitor-transfected HemECs (*P < *0.05) (Fig. 3A). We also detected changes in FGFR1 protein expression in HemECs after transfection with miR-424 mimic or inhibitor using western blot analysis. Similarly, the FGFR1 protein expression in miR-424 mimic-transfected HemECs was significantly lower than in the control mimic-transfected group, while the FGFR1 protein expression in miR-424 inhibitor-transfected HemECs was significantly higher than in the control inhibitor-transfected group (*P* < 0.05) (Fig. 3B,C). Our results indicated that overexpression of miR-424 significantly downregulated FGFR1 expression in HemECs, while miR-424 inhibition significantly upregulated FGFR1 expression in HemECs.

As miRNA mimic or inhibitor can easily have “off-target” effects on closely related miRNAs within a family, we assessed the expression of other family members (miR-15a, miR-15b and miR-16) in HemECs before and after transfection with miR-424 mimic or inhibitor (Fig. 3D). The results demonstrated that transfecting HemECs with miR-424 mimic or inhibitor did not alter the expression levels of miR-15a, miR-15b or miR-16. This indicated that in the miR-15/107 family, miR-424 exert a mainly role in HemECs.

### FGFR1 was a target of miR-424

**Figure 4:**
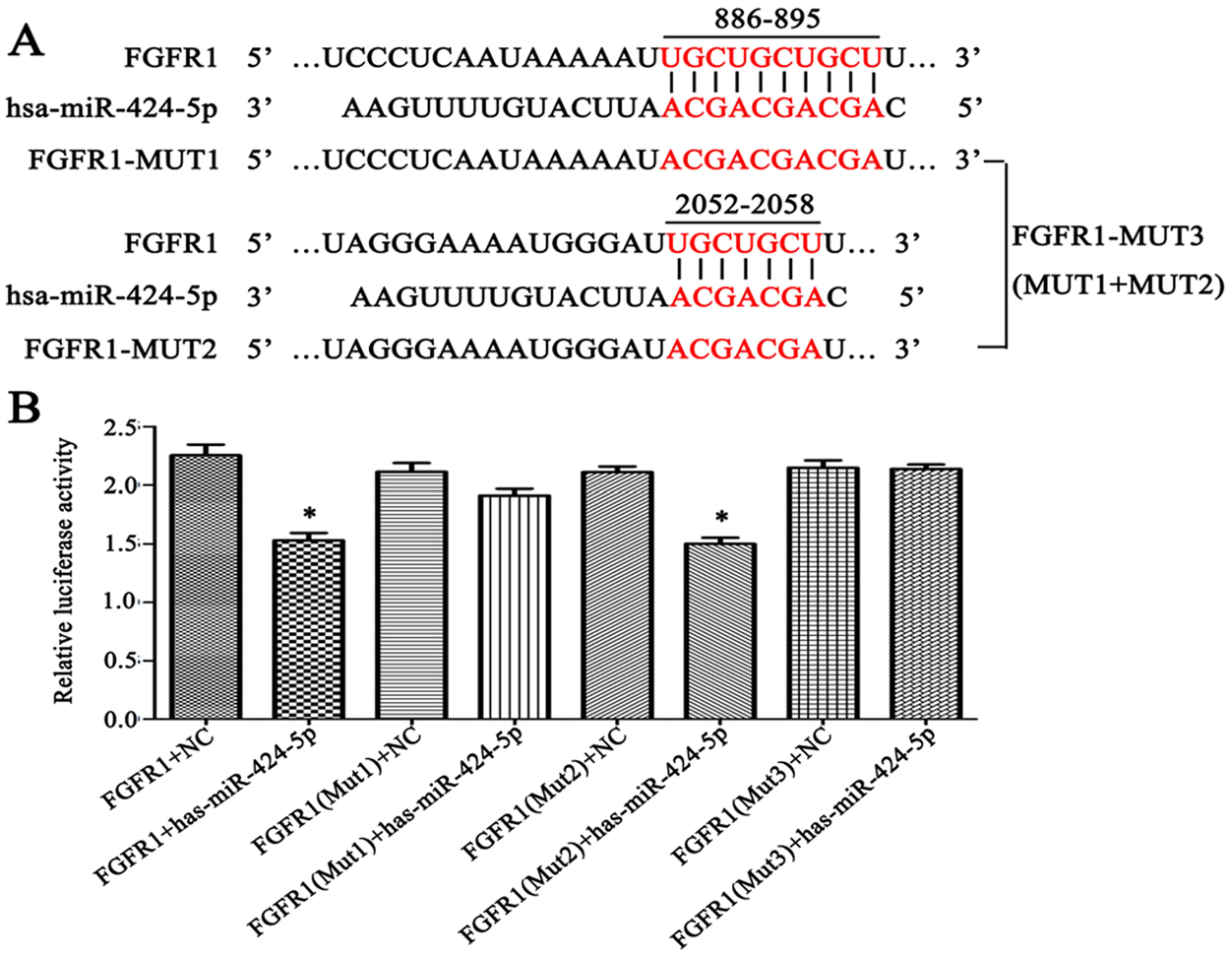
FGFR1 is a target of miR-424. (**A**) MicroRNA target gene prediction software was used to predict the possible existence of miR-424 binding sites in the FGFR1 mRNA sequence. (**B**) A luciferase reporter analysis suggested that the FGFR1 gene was indeed a target of miR-424. *P < 0.05 was considered to have statistically significant differences.

First, we predicted the target genes of miR-424 using miRNA target gene prediction software. Three miR-424 binding sites were found in the 3′-UTR of the FGFR1 mRNA sequence, including two strong sites (886–892 and 889–895, which can be regarded as one binding site due to overlap) and a weak site (2052–2058) (Fig. 4A). To further confirm this prediction, a luciferase reporter assay was used to evaluate the effect of mutation of these binding sites on gene expression. Consistent with our prediction, the results revealed that the relative luciferase activity of a plasmid containing the wild-type 3′-UTR of FGFR1 was significantly reduced after cells were transfected with miR-424 mimic; mutating the strong binding sites (FGFR1-MUT1) significantly suppressed this effect, while mutating the weak binding site (FGFR1-MUT2) had no significant effect, and mutating all three binding sites (FGFR1-MUT1 + FGFR1-MUT2) completely inhibited the decrease in the relative luciferase activity of the plasmid containing the 3′-UTR of FGFR1. This suggested that FGFR1 was indeed a target gene of miR-424 (Fig. 4B).

### miR-424 modulated HemEC proliferation through bFGF/FGFR1

**Figure 5:**
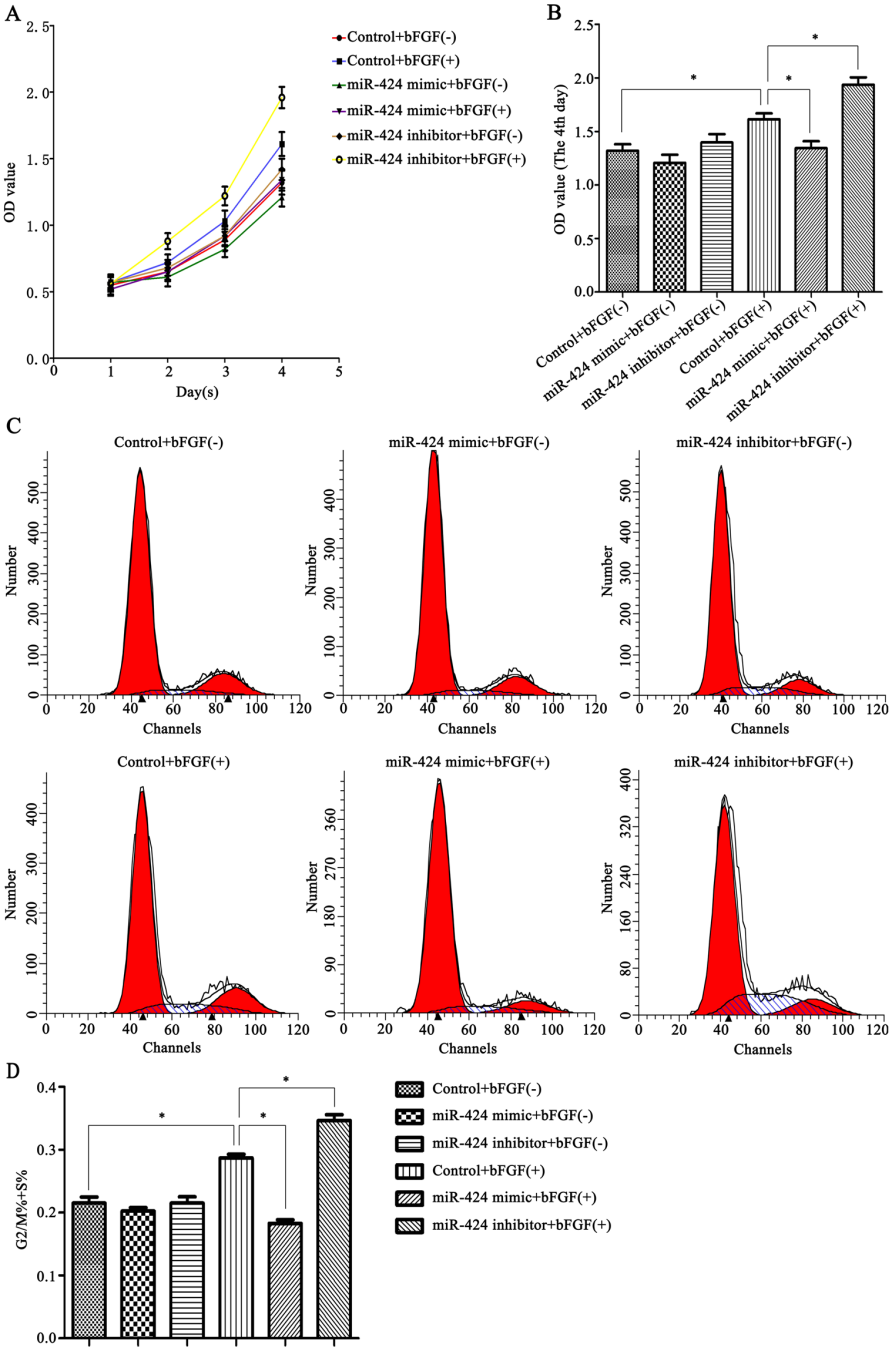
miR-424 modulated HemEC proliferation through bFGF/FGFR1. (**A**,**B**) A CCK-8 assay was used to determine changes in the proliferation capability of HemECs transfected with miR-424 mimic or inhibitor and the mechanism involved. (**C**,**D**) Flow cytometry was performed to examine changes in the cell cycle distribution of HemECs after transfection with miR-424 mimic or inhibitor. *P < 0.05 indicated statistically significant differences.

To elucidate the effect of miR-424 on HemEC proliferation and the mechanism involved, the CCK-8 method was used to determine changes in cell proliferation after cells were transfected with miR-424 mimic or inhibitor. First, the cells were cultured in Endothelial Cell Medium (ECM) (with or without 80 ng/ml bFGF) for one, two, three or four days. Then, CCK-8 reagent was added, and the cells were incubated for another 1 h, and the OD value was measured. Our study found that bFGF stimulation enhanced the ability of HemECs proliferation compared with the control group; under bFGF stimulation, miR-424 overexpression significantly inhibited cell proliferation compared to the control group, while miR-424 inhibition significantly enhanced proliferation of HemECs (*P < *0.05) (Fig. 5A,B). The mechanism may be as follows: bFGF exerts its biological functions in combination with FGFR1. Transfection of HemECs with miR-424 mimic reduced the FGFR1 levels and therefore inhibited the bFGF/FGFR1 pathway, suggesting that miR-424 overexpression inhibited cell proliferation by inhibiting the bFGF/FGFR1 pathway, while miR-424 inhibition enhanced cell proliferation by enhancing the bFGF/FGFR1 pathway.

To further verify this result, flow cytometry was performed to examine changes in the cell cycle after cells were transfected with miR-424 mimic or inhibitor (Fig. 5C,D). The percentage of cells in G2/M and S phases (G2/M% + S%) represented cell proliferation capability. Our results showed that bFGF can extend the G2/M + S phases of the cells; under bFGF stimulation, the G2/M + S phases were significantly shortened in miR-424 mimic-transfected HemECs compared to the control group, while G2/M + S phases were significantly prolonged after cells were transfected with miR-424 inhibitor (*P* < 0.05). Thus, the flow cytometry results also indicated that miR-424 overexpression can significantly decrease HemECs proliferation, whereas inhibition of miR-424 expression can significantly enhance cell proliferation.

### miR-424 modulated HemEC migration through bFGF/FGFR1

**Figure 6:**
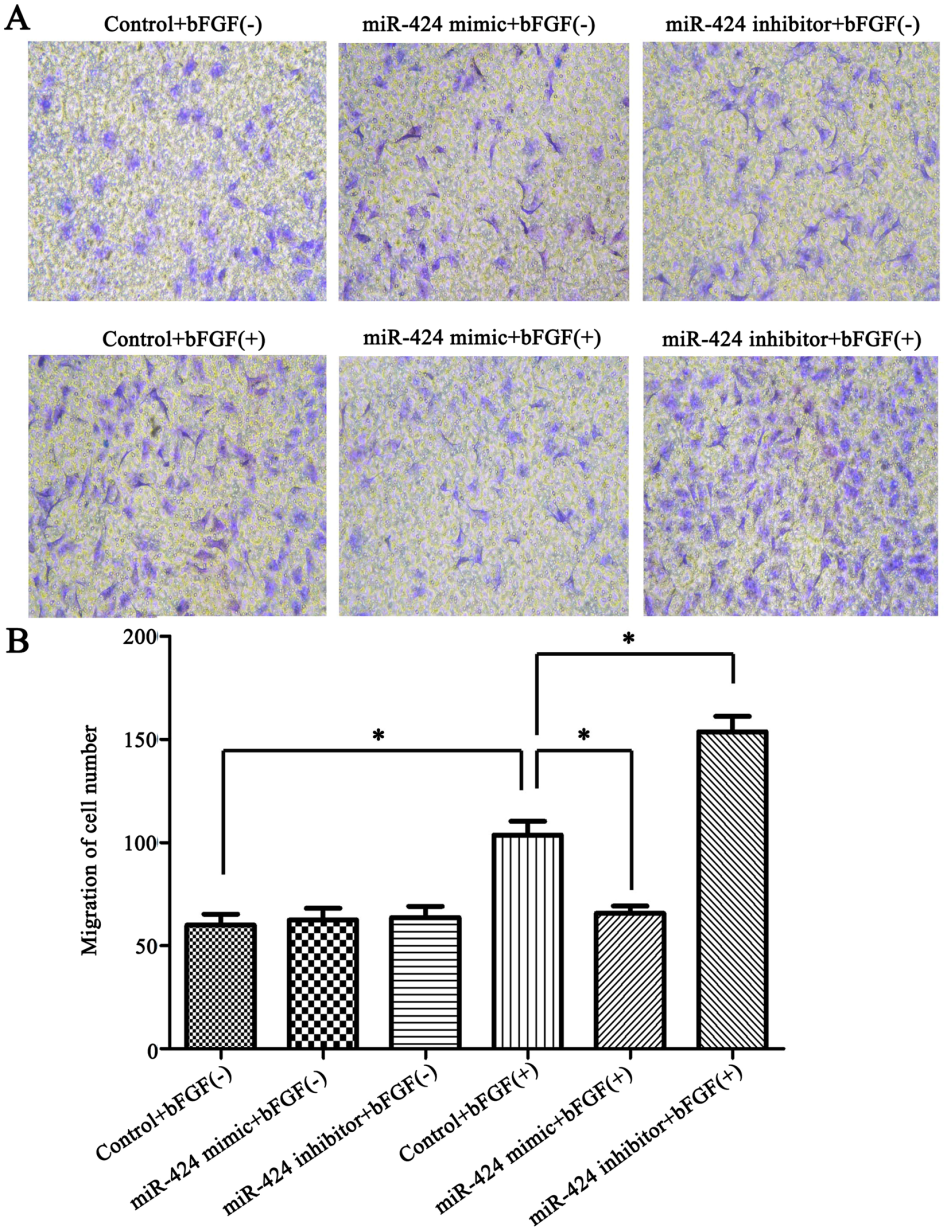
miR-424 modulated HemEC migration through bFGF/FGFR1. (**A**) The changes in HemEC migration capability after transfection with miR-424 mimic or inhibitor (with or without bFGF stimulation). (**B**) Statistical analysis of the quantified migration assay in each group. *P < 0.05 represented statistically significant differences.

A transwell migration assay was used to determine whether miR-424 could affect the migration capability of HemECs. The cells were grouped according to the method described in Materials and Methods. Our results showed that bFGF stimulation enhanced the migration capability of HemECs compared with the control group; under bFGF stimulation, miR-424 overexpression significantly inhibited cell migration compared to the control group, while miR-424 inhibition significantly enhanced cell migration of HemECs (*P < *0.05) (Fig. 6A,B). This result suggested that miR-424 overexpression inhibited cell migration by inhibiting the bFGF/FGFR1 pathway, while miR-424 inhibition enhanced cell migration by enhancing the bFGF/FGFR1 pathway.

### miR-424 modulated HemEC angiogenesis through bFGF/FGFR1

**Figure 7:**
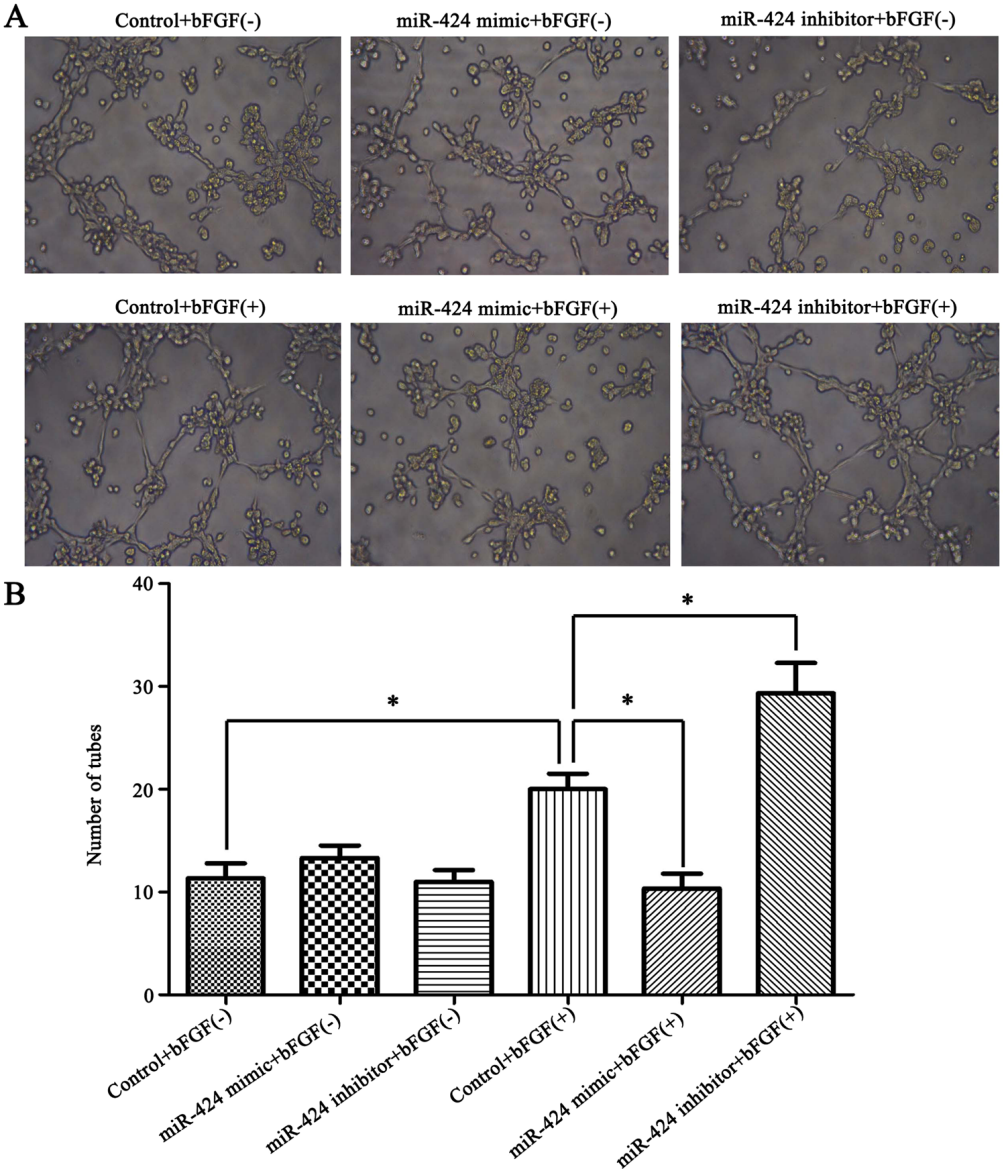
miR-424 modulated HemEC angiogenesis through bFGF/FGFR1. (**A**) The changes in HemEC angiogenesis capability after transfection with miR-424 mimic or inhibitor (with or without bFGF stimulation). (**B**) Statistical analysis of the quantified tubes formation assay in each group. *P < 0.05 represented statistically significant differences.

To study the effect of miR-424 on HemEC angiogenesis and the mechanism involved, we conducted a tube formation assay. The results were consistent with the above-mentioned experimental results. Our results revealed that bFGF stimulation enhanced the tube formation capability of HemECs compared with the control group; under bFGF stimulation, miR-424 overexpression significantly inhibited tube formation compared to the control group, while miR-424 inhibition significantly enhanced tube formation of HemECs (*P < *0.05) (Fig. 7A,B). This result indicated that miR-424 overexpression inhibited HemEC angiogenesis by inhibiting the bFGF/FGFR1 pathway, while miR-424 inhibition enhanced angiogenesis by enhancing the bFGF/FGFR1 pathway.

### miR-424 regulated the ERK1/2 phosphorylation level through bFGF/FGFR1 signaling

**Figure 8:**
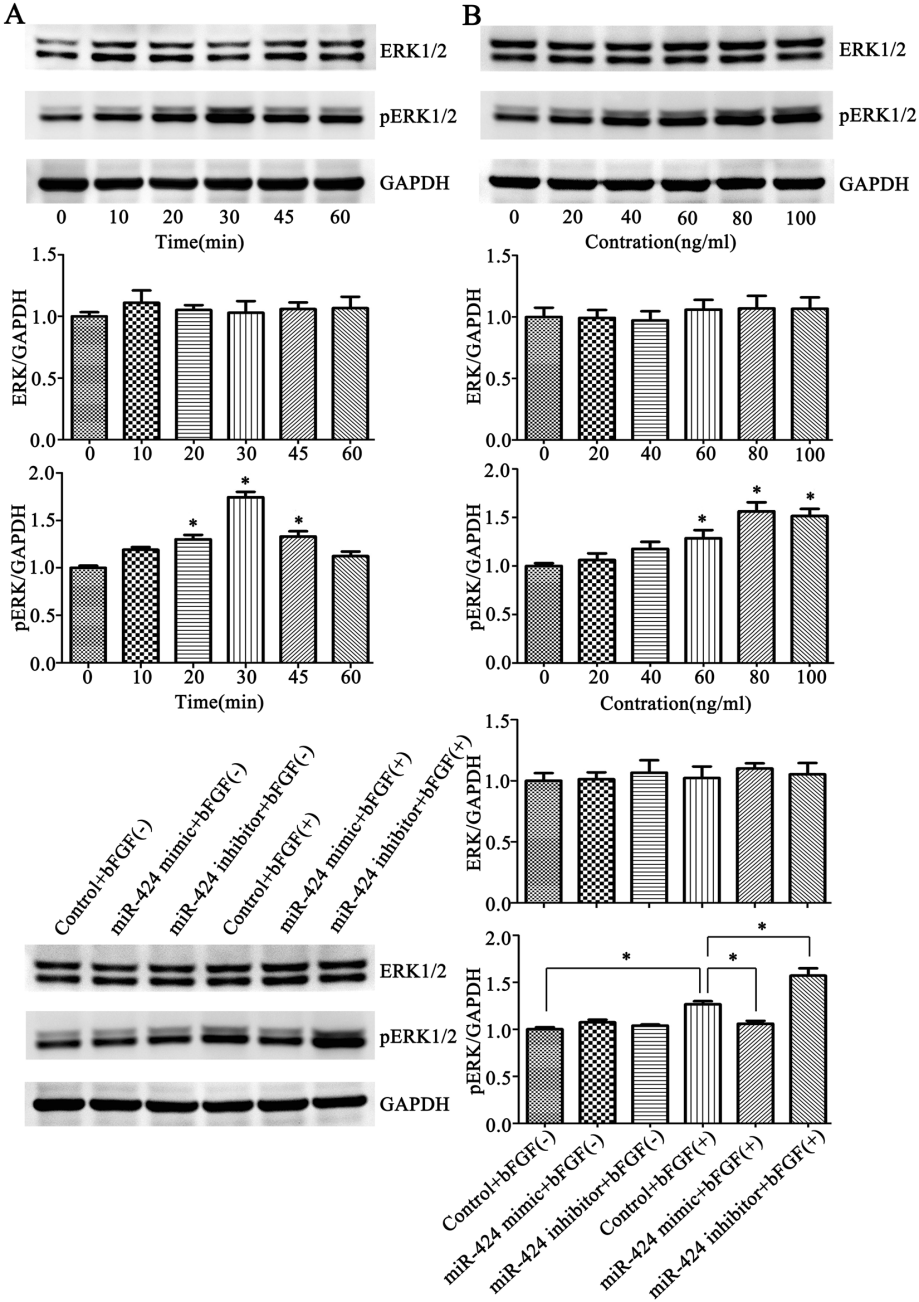
miR-424 regulated the ERK1/2 phosphorylation level through bFGF/FGFR1 signaling. (**A**) ERK1/2 phosphorylation levels were detected after HemECs were stimulated with 80 ng/ml bFGF for different lengths of time. (**B**) ERK1/2 phosphorylation levels were detected after HemECs were stimulated with different concentrations of bFGF for 30 min. (**C**) ERK1/2 phosphorylation levels were detected after miR-424 mimic or inhibitor-transfected HemECs were stimulated with or without 80 ng/ml bFGF for 30 min. *P < 0.05 indicated statistically significant differences.

To directly elucidate the role of miR-424 in the bFGF/FGFR1 signaling pathway, western blot analysis was performed to detect the effect of miR-424 overexpression or inhibition on ERK1/2 phosphorylation. First, HemECs were stimulated with 80 ng/ml bFGF for different lengths of time (0, 10, 20, 30, 45 or 60 min). The results revealed that the ERK1/2 phosphorylation level increased gradually within 30 min, peaked at 30 min, and then gradually decreased (*P* < 0.05) (Fig. 8A). Then, the HemECs were stimulated with different concentrations of bFGF (0, 20, 40, 60, 80 or 100 ng/ml) for 30 min. We found that the highest ERK1/2 phosphorylation level was observed upon treatment with 80 ng/ml bFGF (*P < *0.05) (Fig. 8B). Finally, HemECs transfected with miR-424 mimic or inhibitor were stimulated with or without 80 ng/ml bFGF for 30 min. The results showed that bFGF stimulation significantly enhanced the ERK1/2 phosphorylation level of HemECs; under bFGF stimulation, miR-424 overexpression significantly inhibited ERK1/2 phosphorylation compared to the control group, while miR-424 inhibition significantly enhanced ERK1/2 phosphorylation in HemECs (*P < *0.05) (Fig. 8C). These results also suggested that miR-424 overexpression inhibited ERK1/2 phosphorylation by inhibiting the bFGF/FGFR1 pathway, while miR-424 inhibition enhanced ERK1/2 phosphorylation in HemECs by enhancing the bFGF/FGFR1 pathway.

## Discussion

Researchers have proposed many theories, including the placental origin theory, transfer doctrine, doctrine of progenitor cells and exogenous factor theory^40^. However, so far, no single theory has explained the clinical, microscopic and molecular characteristics of hemangiomas. For several decades, IH was considered to arise from dysregulated differentiation of embryonic cells. Greenberger *et al*. isolated a primitive mesenchymal cell from proliferative IH using anti-CD133-coated magnetic beads. When CD133-selected cells (hemangioma stem cells, HemSCs) were sub-cutaneously implanted into nude mice, the HemSCs formed GLUT1+ vessels (a specific marker of IH) within 7–14 days^41^. Moreover, these cells can differentiate into endothelium, adipocytes and pericytes^41,42^. These data strongly suggested that vasculogenesis was an important mechanism underlying hemangioma-genesis^43^.

Abnormal regulation of angiogenesis is considered to play a key role in the pathogenesis of hemangioma. The transition from the proliferative phase to the involuting phase is a gradual process of proliferation and apoptosis disequilibrium in endothelial cells, which results from an imbalance between vascular endothelial cell growth promoting factors and inhibiting factors^44^. Therefore, vascular endothelial growth factors play important roles in the proliferation and regression of hemangioma. bFGF and VEGF, which are considered to be the most effective and specific angiogenic factors^11,45^, are essential in angiogenesis and angiopoiesis^46,47^.

Previous studies have shown that miR-424 often serves as a tumor suppressor gene^36^. In addition, recent studies have shown that miR-424 can regulate VEGF and bFGF signaling in human umbilical vein endothelial cells (HUVECs) by targeting VEGF, VEGFR2 and FGFR1, and thereby inhibit endothelial cell proliferation, migration, tube formation and angiogenesis. Conversely, another study revealed that miR-424 promoted angiogenesis in hypoxic endothelial cells, indicating that miR-424 might have an environment-dependent effect^48,49^. Kim *et al*. noted that miR-424 and miR-503 can exert anti-proliferative effects by targeting bFGF and FGFR1 in pulmonary arterial hypertension (PAH)^50^. Nakashima *et al*. reported that downregulation of miR-424 contributed to abnormal cell proliferation and angiogenesis via MEK1 and Cyclin E1 in senile hemangioma^39^. MEK1 and Cyclin E1 belong to the same signaling pathway, which regulates cell cycle progression. MEK1 is known as an upstream molecule of ERK and one of the most important mitogenic regulators^51^. Cyclin E1 is the downstream target of ERK, which is also implicated in cell cycle regulation^52,53^. Nakashima *et al*. first demonstrated the role of miR-424 in angiogenic processes and identified MEK1 and Cyclin E1 as the target genes of miR-424^39^. However, the role of miR-424 in infantile skin hemangioma is still unclear. Our results showed that miR-424 expressed at low levels in infantile skin hemangioma tissues and was negatively correlated with the expression of FGFR1; miR-424 overexpression downregulated the expression of FGFR1 in HemECs, while miR-424 inhibition upregulated FGFR1 expression. In addition, luciferase reporter analysis confirmed that FGFR1 was a target gene of miR-424.

Many studies have reported that HemECs stagnate in the early developmental stages of differentiation^54,55^. A cell cycle analysis performed by Kim *et al*. revealed that miR-424 and miR-503 overexpression can induce cell cycle arrest at the G0/G1 phases in both normal pulmonary artery endothelial cells (PAECs) and PAECs under pulmonary arterial hypertension (PAH). In addition, cell migration assays showed that miR-424 and miR-503 overexpression reduced PAEC migration^50^. Our flow cytometry analysis indicated that miR-424 overexpression reduced the percentage of HemECs in G2/M + S phases, which was consistent with the previous reports demonstrating early cell cycle arrest^54,55^; indicating that miR-424 overexpression significantly decreased the proliferation of HemECs. Furthermore, CCK-8, Transwell migration and tubule formation assays revealed that miR-424 overexpression significantly inhibited HemEC proliferation, migration and angiogenesis by inhibiting the bFGF/FGFR1 pathway; while miR-424 inhibition enhanced HemEC proliferation, migration and angiogenesis.

Extracellular signal-regulated kinase 1/2 (ERK1/2) is an important signal transduction molecule which belongs to the mitogen-activated protein kinase (MAPK) signal transduction pathway family. Its role is to conduct extracellular signals into cells, causing a series of biological effects^56^. ERK1/2 is a key downstream target of bFGF/FGFR1 signaling in endothelial cells. The ERK1/2 phosphorylation level may reflect the activation status of the bFGF/FGFR1 signaling pathway^57^. To illuminate the role of miR-424 in the bFGF/FGFR1 signaling pathway directly, we examined the effect of miR-424 overexpression or inhibition on ERK1/2 phosphorylation. Our results showed that miR-424 overexpression significantly inhibited ERK1/2 phosphorylation in HemECs, while miR-424 inhibition significantly enhanced ERK1/2 phosphorylation, suggesting that miR-424 may suppress the bFGF/FGFR1 pathway, thereby inhibiting ERK1/2 phosphorylation, and thus affecting its function in HemECs.

In short, this is the first study to investigate the expression, function and possible mechanism of miR-424 in infantile skin hemangioma. Our results indicated that miR-424 suppressed the bFGF/FGFR1 pathway by targeting FGFR1, thereby inhibiting ERK1/2 phosphorylation, and thus inhibiting cell proliferation, migration and tube formation and the development of infantile skin hemangioma. Therefore, miR-424 may serve as an alternative or supplementary approach to classic therapeutic strategies in high-risk IH in the future. Treatment of angiogenesis and tumor growth in IH with miR-424 may provide a new path for clinical and basic scientific hemangioma research.

## Materials and Methods

### Tissue specimen collection

Paraffin-embedded tissue specimens from 52 cases of infantile skin hemangioma and 14 cases of normal subcutaneous tissue surrounding hemangiomas were collected in 2013–2014 from the Institute of Pathology of Tongji Hospital, Huazhong University of Science and Technology. Of the 52 cases, proliferative and involuting hemangioma accounted for 26 cases respectively. Informed consent was obtained from each patient.

The proliferative phase and involuting phase were determined according to the WHO standards. Proliferative lesions presented mainly as small round luminal-like capillaries and were composed of endothelial cells and vascular pericytes that were rich in cytoplasm and contained enlarged nuclei. The capillaries showed a slightly lobular arrangement and were segmented by normal tissues or slender fibrous intervals. Moreover, they had a wealth of arterial and venous blood supplies and mitotic figures were common. In the involuting phase, the endothelial cells and vascular pericytes became flat, the lumen enlarged and the mitotic figures disappeared. Finally, the capillaries gradually disappeared and were replaced by loose connective tissue^58^.

### Cell culture and cell transfection

HemECs were kindly provided by Biossci Biotechnology (Hubei, China). The miR-424 mimic and miR-424 inhibitor were synthesized by GenePharma (Shanghai, China). The cells were conventionally recovered in ECM (Sciencell, USA) and incubated in a 37 °C, 5% CO_2_ incubator. Then, the HemECs were seeded in 6-well plates at a density of 2 × 10^6^ cells/ml per well to reach 60% confluency the next day. Before transfection, 100 pmol of miR-424 mimic (or control mimic, miR-424 inhibitor or control inhibitor) was added to 250 μl of OPTI-MEM to generate solution A. Meanwhile, 5 μl of Lipofectamine 2000 was added to 250 μl of OPTI-MEM to make solution B. After 5 min of incubation, solutions A and B were mixed at room temperature for 20 min to form solution C. Then, 800 μl of OPTI-MEM was added to each well. Finally, solution C was added dropwise into each well to ensure that the miR-424 mimic or miR-424 inhibitor reached a final concentration of 80 nM. After 4–6 h, the medium was replaced with fresh ECM. The following experiments were all performed 48 h after transfection.

### Immunohistochemistry

The paraffin-embedded tissue specimens from 52 cases of infantile skin hemangioma and 14 samples of normal subcutaneous tissue surrounding hemangiomas were cut into 3-μm slices. After deparaffinization and rehydration, the antigens were retrieved using a high-pressure method with sodium citrate buffer (pH 6.0) for 1.5 min. Then, 3% H_2_O_2_ was added to inactivate the endogenous peroxidase activity at room temperature for 10 min, followed by blocking of nonspecific antigens with 5% BSA for 1 h. Subsequently, the specimens were incubated with an anti-FGFR1 antibody (Proteintech, 60325-1-Ig, 1:800) at 4 °C overnight and then incubated with the secondary antibody working solution (Dako, GK500705) for 1 h at 37 °C the next day. Finally, specimens were stained with a DAB Substrate Kit (Invitrogen, USA) for 5 min.

### Real-Time PCR

Total RNA was extracted from paraffin-embedded specimens with a paraffin-embedded tissue microRNA rapid extraction kit (BioTeke, China). TRIzol reagent (Invitrogen, USA) was used to extract total cellular RNA. For cDNA synthesis, 2 μg of RNA was added to a 20 μl reverse transcription reaction as a template. The PCR primers were as follows: FGFR1 sense primer, ACCCCGCCAGGACCCGAACA and anti-sense primer, GACCAGCACAGCCCAGAAGA; GAPDH sense primer, AGGTCGGAGTCAACGGATTTG and anti- sense primer, GTGATGGCATGGACTGTGGT^59^. For miR-424 detection, U6 snRNA was used as the endogenous control. Real-time PCR was performed using an ABI 7500 real-time instrument (ABI StepOne Plus, USA) according to the manufacturer’s protocol. The 20 μl reactions included 10 µl of All-in-One^TM^ qPCR Mix, 0.4 μl of the sense primer, 0.4 μl of the anti-sense primer and 5 μl of cDNA. The samples were subjected to an initial denaturation at 95 °C for 10 min, followed by 40 amplification cycles of denaturation at 95 °C for 10 s, annealing at 60 °C for 20 s and extension at 72 °C for 20 s.

### Western blot analysis

After 48 h of transfection, the cells were stimulated with bFGF. Then, the total proteins were extracted using RIPA lysis buffer, and the protein concentrations were determined with a Bradford Protein Assay kit (Beyotime, China). Thereafter, equal amounts of protein were electrophoresed on SDS-PAGE gels and transferred to PVDF membranes, followed by blocking with 5% BSA for 1 h. Then, the membranes were incubated with an FGFR1 antibody (1:800, Proteintech, USA), GAPDH antibody (1:10000, Sungene Biotech, China), ERK1/2 antibody (1:1000, Bioworld, USA) or phospho-p44/42 MAPK (ERK1/2) (Thr202/Tyr204) antibody (1:1000, Cell Signaling Technology, USA) at 4 °C overnight. The next day, after washing with TBST, the membranes were incubated with goat anti-rabbit IgG/HRP (1: 20000, Sungene Biotech, China) and goat anti-mouse IgG/HRP (1: 10000, Sungene Biotech, China) at 37 °C for 1 h. Finally, the signal was detected using ECL reagents (Beyotime, China).

### Dual luciferase reporter analysis

A target gene predictive analysis revealed that there were potential miR-424 binding sites in the 3′-UTR of the FGFR1 mRNA sequence (http://www.microrna.org). To confirm this hypothesis, a luciferase reporter analysis was performed. First, we designed and amplified the 3′UTR of FGFR1. The amplification primers were as follows: FGFR1-UTR-F, CAGGGGAGGATTCCGTCT and FGFR1-UTR-R, GAGTCAGGTCAATTTCACTGTCTTT (Biossci, China). Then, the amplified products were inserted into a psiCHECK™-2 Vector (Promega, C8021, USA) at an XhoI site to construct a psiCHECK™-2-FGFR1-3’UTR plasmid. The linker-adapter primers were as follows: FGFR1-check2 (XhoI) -F, TAGGCGATCGCTCGAGCTGCCACCCACACG and FGFR1-check2 (XhoI) -R, AATTCCCGGGCTCGAGTCAGGTCAATTTCAC (Biossci, China). Finally, the miR-424 binding sites in the FGFR1 3’-UTR were mutated. The mutagenic primers were as follows: FGFR1-Mut1-F, ATACGACGACGATCATTTATCTATGGGCT and FGFR1-Mut1-R, TGATCGTCGTCGTATTTTTATTGAGGGACCTAAAC; FGFR1-Mut2-F, GGATACGACGATTAAATTTCTGAGCTAGGGAT and FGFR1-Mut2-R, TTAATCGTCGTATCCCATTTTCCCTACAGAA. The day before transfection, the cells were plated in 24-well plates at a density of 5 × 10^4^ cells/well. The next day, the plasmids (wild-type and mutant) were co-transfected with miR-424 mimic or inhibitor into HemECs. After 48 h of transfection, the luciferase activities were measured using a Dual-Glo Luciferase Assay System (Promega, E2920, USA) according to the manufacturer’s protocol.

### CCK-8 proliferation assay

For the cell proliferation assay, transfected HemECs were digested and resuspended in ECM (with or without 80 ng/ml bFGF) at a cell density of 2 × 10^4^ cells/ml. Then, 100 μl of the cell suspension was seeded into each well of 96-well plates. After culturing for one, two, three or four days, the cells were treated with 10 μl of CCK-8 reagent (Beyotime, China) and incubated at 37 °C for 1 h. The absorbance was measured at 450 nm, and the growth curves were constructed based on OD values.

### Flow cytometry

After digestion and centrifugation, transfected HemECs (with or without bFGF stimulation for 24 h) were treated with 2 ml of ice-cold 70% ethanol, repeatedly pipetted into a single-cell suspension and fixed at −20 °C for 24 h. After that, the cells were treated with 20 μg/ml RNaseA (Invitrogen, USA) for 1 h at 37 °C and then stained in the dark with 50 μg/ml PI (Beyotime, China) for 30 min at room temperature. The cell cycle distribution was determined by flow cytometry (BD Biosciences, USA) at 488 nm, and the fitting analysis was performed using DNA ModFit software.

### Transwell migration assay

After 48 h of transfection, HemECs (2 × 10^5^ cells /ml) were seeded in 24-well 8.0 μm transwell inserts. Then, 400 μl of ECM (control group, miR-424 mimic group and miR-424 inhibitor group) or 400 μl of ECM containing 100 ng/ml bFGF (bFGF + control group, bFGF + miR-424 mimic group and bFGF + miR-424 inhibitor group) was added into each lower chamber. In the upper chambers, 100 μl of control mimic-transfected HemECs (control group, bFGF + control group), miR-424 mimic-transfected HemECs (miR-424 mimic group, bFGF + miR-424 mimic group) or miR-424 inhibitor-transfected HemECs (miR-424 inhibitor group, bFGF + miR-424 inhibitor group) were correspondingly added to individual wells. After 24 h of culture, the cells that had relocated to the transwell chamber base film were fixed with 4% paraformaldehyde and stained with 0.1% crystal violet (Beyotime, China). The cells were counted in five fields (the middle and surrounding visions) under × 200 magnification, and the results were then averaged.

### Tube formation assay

Matrigel was melted in a 4 °C ice bath overnight. Then, 50 μl of Matrigel was added to each well of prechilled 96-well plates (performed on ice) and incubated at 37 °C for 30–60 min. The transfected HemECs were resuspended in low-serum medium (2% FBS) at a density of 2.5 × 10^4^ cells/ well and seeded into the Matrigel-covered 96-well plates. Then, 80 ng/ml bFGF was added to the bFGF stimulation group, and the cells were incubated at 37 °C for 2–8 h. Finally, the tubules were counted in five fields (the middle areas of the well and the surrounding areas) under ×200 magnification and photographed.

### Statistical analysis

All the experimental data are presented as the mean ± SD. The differences between variables were analyzed using one-way ANOVA or a chi-square test. *P* < 0.05 was considered to indicate statistically significant differences.

### Ethical approval and informed consent

All experiments were carried out in accordance with relevant guidelines and regulations. For human tissue samples, approval was obtained from the Medical Ethics Committee of Tongji Hospital, Tongji Medical College, Huazhong University of Science and Technology. In addition, informed consent was obtained from each patient.
